# Taxonomic and functional anuran beta diversity of a subtropical metacommunity respond differentially to environmental and spatial predictors

**DOI:** 10.1371/journal.pone.0214902

**Published:** 2019-11-14

**Authors:** Diego Anderson Dalmolin, Alexandro Marques Tozetti, Maria João Ramos Pereira

**Affiliations:** 1 Programa de Pós-Graduação em Biologia Animal, Universidade Federal do Rio Grande do Sul, Porto Alegre, Rio Grande do Sul, Brasil; 2 Universidade do Vale do Rio dos Sinos, São Leopoldo, Rio Grande do Sul, Brazil; 3 Centre for Environmental and Marine Studies, Universidade de Aveiro, Aveiro, Portugal; Universitat Trier, GERMANY

## Abstract

Anurans exhibit limited dispersion ability and have physiological and behavioural characteristics that narrow their relationships with both environmental and spatial predictors. So, the relative contributions of environmental and spatial predictors in the patterns of taxonomic and functional anuran beta diversity were examined in a metacommunity of 33 ponds along the coast of south Brazil. We expected that neutral processes and, in particular, niche-based processes could have similar influence on the taxonomic and functional beta diversity patterns. Distance-based methods (db-RDA) with variation partitioning were conducted with abundance data to examine taxonomic and functional facets and components (total, turnover and nestedness) in relation to environmental and spatial predictors. Processes determining metacommunity structure differed between the components of beta diversity and among taxonomic and functional diversity. While taxonomic beta diversity was further accounted by both environmental and spatial predictors, functional beta diversity responded more strongly to spatial predictors. These two contrasting patterns were different to what we had predicted, suggesting that while there is a taxonomic turnover mediated by environmental filters, the spatial distance promotes the trait dissimilarity between sites. In addition, our data confirm that neutral and niche-based processes operate on anuran metacommunities even at short geographic scales. Our results reinforce the idea that studies aiming to evaluate the patterns of structure in metacommunities should include different facets of diversity so that better interpretations can be achieved.

## Introduction

Beta diversity connects the spatial structure of communities to a variety of ecological processes, such as neutral processes (e.g. dispersion limitation) and niche-based processes (e.g. limiting similarity and environmental filtering) [1, 2, 3, 4]. Beta diversity represents the amount of variation in species composition between a set of local communities [2, 5, 6], and can be divided into two additive components: spatial turnover and nestedness-resultant [7, 8]. Spatial turnover occurs when some species are replaced by others as a result of ecological processes (e.g. environmental filtering and dispersion limitation) that restrict their occurrence in certain places [2, 8, 9]. Nestedness, on the other hand, occurs when non-random processes of species loss result in the ordered deconstruction of assemblages, leading to the formation of local sets poorer in the number of species, and subsets of richer sites [9, 10, 11]. Despite their distinct nature, these two components are complementary and main drivers of dissimilarity patterns between communities [7].

Within the metacommunity theory, the organization of local assemblies is thought to occur at broader spatial scales [12, 13]. In metacommunities structured by neutral processes, the dispersion limitation and demographic stochasticity are the dominant factors, so that geographical distance between communities is the best predictor of beta diversity [14, 15]. In contrast, ecological interactions and environmental conditions are the most important factors in metacommunities structured by niche processes, and the environmental distance between communities should be the best predictor of beta diversity [12, 16]. However, several studies suggest that the action of these processes is not mutually exclusive, and that the structuring of biological metacommunities results from the interaction of the two processes–neutral and niche-based [13, 17]. Indeed, in aquatic metacommunities deterministic processes (especially environmental filtering) seem to be dominant, though neutral processes also contribute to the observed patterns of beta diversity [9].

Taking into account that biological communities result from a complexity of interactions between organisms, environment and space, the incorporation of functional traits (functional diversity) in community and metacommunity studies has been widely advocated [18, 19]. In fact, the use of an integrative approach where taxonomic and functional diversities are taken into account is advantageous. First, the evaluation of communities using only taxonomic identity is often difficult to interpret, since taxonomic groups may contain phylogenetic and ecological lineages in conflict between convergence and adaptive divergence [20, 21, 22]. In addition, the functional approach allows elucidating the ‘true role’ of each species in ecosystem processes and their resistance and resilience to environmental changes [23, 24]. Finally, several studies found congruent responses of the two metrics of diversity for the same ecosystem processes [19, 25, 26], although these relationships may vary according to the taxonomic group of interest [27, 28].

Neotropical anurans are considered excellent ecological models because they are locally abundant and sampling of most groups is relatively easy. Anurans have highly permeable skin, a complex and biphasic life cycle, limited dispersion, and geographically restricted distribution patterns [29, 30]. So, compositional variation in anuran seems to result from several factors, such as available area and hydroperiod, vegetation cover, type of surrounding matrix and geomorphology [26, 31, 32, 33, 34]. Thus, both environment and space tend to strongly contribute to the patterns of taxonomic and functional dissimilarity between anuran communities [30, 35, 36].

Although anuran beta diversity has already been addressed in several studies [33, 36, 37], few have used an integrative approach to describe patterns of anuran beta diversity [30]. Although the dominance of environmental predictors over beta diversity has been reported in several studies [26, 29, 37], others have reported considerably greater spatial effects [38], or even a balance between both [30, 33]. These discrepancies occur because the different characteristics of anurans' life histories respond differently to ecological predictors, so that the metacommunity structure may differ between groups occurring in the same region [39]. Thus, the concomitant evaluation of taxonomic and functional information can resolve much of the mismatch about the anuran metacommunity structure and provide important information for the conservation of species and their functions in community and ecosystem properties [40].

In this study we investigated the relationship between environmental and spatial components and the patterns of taxonomic and functional beta diversity in a metacommunity of anurans from the coastal subtropical region of southern Brazil. We expect beta diversity to be influenced by both environmental and spatial components, with a greater contribution of the environmental component to the distribution of traits and species [41]. We also expect diversity components–taxonomic and functional–to present similar responses to the sets of descriptors evaluated [30]. Through functional traits, species can shape, change and accommodate in the environment where they occur [42, 43]. Consequently, species distribution can be expected as resulting from combinations of ecologically relevant characteristics allowing them to persist in a given set of environments [44]. We thus expect functional diversity to be a better indicator of the ecological processes responsible for the structuring of the anuran metacommunity [41, 45].

## Material and methods

### Ethics statement

Collection permits were provided by Instituto Chico Mendes de Conservação da Biodiversidade (ICMBio) (authorization 55409). Field studies did not involve endangered or protected species. We restricted manipulation of animals in the field to minimal as we sampled just specimens restricted in the collection units (see the section 2.3). Specimens collected were identified, measured and immediately released in the same pond where they were sampled. All sampling procedures were reviewed and specifically approved as part of obtaining the field permits by ICMBio (see above).

### Study area

**Fig 1:**
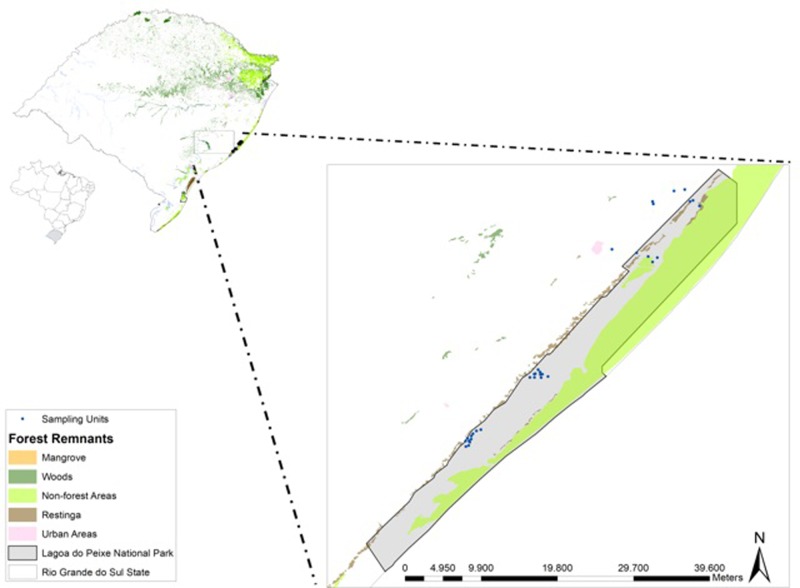
Study area at Lagoa do Peixe National Park, southern Brazil. Sampled ponds are represented by blue circles.

This study was done in Lagoa do Peixe National Park (PNLP; 31°02’-31°48’S; 50°77’-51°15’W; Fig 1), the only Ramsar site in southern Brazil [46]. With a length of 64 km and an average width of 6 km, the PNLP comprises over 34,000 hectares of protected wetlands, integrating the Coastal Plain of the State of Rio Grande do Sul, one of the regions of southern Brazil with higher concentration of wetlands [47]. The climate is subtropical humid, and temperatures range between 13°C and 24°C with annual average of 17.5°C. The mean annual precipitation varies between 1200 and 1500 mm [48]. The vegetation along water bodies is typical of wetlands, with a predominance of tree and grass vegetation around water bodies and aquatic macrophytes at the edges and inland.

### Anuran surveys and trait measurement

We sampled adult anurans in 33 water bodies throughout the study region (Fig 1). Ponds were selected based on biotic and abiotic characteristics (see Table A in S1 File), insulation of other ponds (sample independence), accessibility and landowner permission. Distances between ponds ranged from 0.7 to 39 km. Sampling was performed monthly, from October 2016 to March 2017. The hydroperiod characterization of each pond was performed monthly by recording total area and average depth. Most of the sampled ponds showed monthly variation in water volume, but remained with over 50% of the initial volume registered (the exceptions were the ponds that showed small reductions). Thus, the ponds were characterized as semi-permanent.

We used both calling surveys and active search at breeding habitats to record the number of calling males of each species in each pond [49]. Samplings were done from 6 p.m. to 0 a.m. The total effort per pond was 1 hour per month, totalling 6 hours of sampling per pond. Sampling was performed by DAD with the help of three additional researchers. DAD was responsible for both accounting for the individuals present in each pond and for measuring the morphological traits of each individual.

We measured five morphological traits for each individual captured: head shape; eyes position; eye size; relative length of limbs; body mass (see Table C in S1 File for more information). Addittionaly, we compiled six life history traits from literature: reproductive mode; relative number of eggs; daily activity period; type of habitat; fossorial habit; reproductive season. These 11 traits were chosed based on perceived importance for determining habitat use and species resilience. All attributes were used to construct a pairwise distance matrix of species. In this procedure we used the Gower standardization for mixed variables [50].

### Environmental and spatial variables

The eight environmental descriptors measured in this study and their description are presented in Table A in S1 File. Area, depth and number of vegetation types around the pond, pond vegetation (inside the pond), margin configuration and pond substrate were measured in the field through visual interpretation in an area around 5 meters from the edge of the ponds. The distance to the nearest forest fragment and distance to the nearest sampled pond were obtained from high-resolution aerial photographs of the region inspected immediately after samplings, available from Google Earth (http://earth.google.com/), combined with field inspection.

We used distance-based Moran’s Eigenvector Maps (dbMEMs) [50, 51] to create spatial variables (eigenvectors) based on the Euclidean distance matrix of the geographical coordinates of the ponds. First of all, we defined the neighbourhood matrix, which describes the spatial relationships among objects [51]. In other words, we defined which ponds are neighbours and which are not. We used as spatial neighbourhood graphs 'Delaunay triangulation', 'Gabriel graph' and 'Minimum spanning trees', and as a weight measure we used the linear distances between ponds. We selected the best neighbourhood matrix based on AICc. The most parsimonious model was the one based on the 'Gabriel graph', and the truncation distance was 18.03 km (AIC = 11.22 versus 12.88 for the null model). This model also generated 23 spatial variables (eigenvectors), eight of which with positive autocorrelation.

### Data analysis

#### Data matrices

We built four matrix types containing the measured data of all ponds: (i) an abundance matrix, which contained the total species count for each pond; (ii) a trait matrix, containing average trait values for each species; (iii) an environmental matrix, containing all environmental descriptors measured in each pond; and (iv) a spatial matrix, containing all dbMEMs.

As suggested by [52], we considered the abundance of each species in a given pond as the abundance of calling males recorded in the month of highest recorded abundance. This procedure prevents underestimates of population abundance caused by calculating the mean of successive samples and prevents overestimates caused by the re-counting of individuals if successive samples are summed [52]. Abundance data were transformed using the Hellinger distance [50] to homogenize variation among species abundances. Environmental descriptors values were standardized by subtracting each value from the average of the descriptor and dividing the result by the standard deviation.

#### Assessing the anuran beta diversity components

We partitioned beta diversity into overall beta diversity, turnover and nestedness components following the methods proposed by [8]. This procedure was performed using the function “beta.pair” in the R package betapart [53] and the function “beta” in the R package BAT [54]. We used the Bray-Curtis dissimilarity for the abundance data. This procedure produced three dissimilarity matrices (Table 1).

**Table 1:** Summary of beta diversity index and their nomenclatures used in this study.

Beta diversity index		Nomenclature
Total	Overall spatial turnover	βBray
Turnover	Turnover immune to species richness variation	βBal
Nestedness	Nestedness resulting from species richness differences between sites	βGra

#### Community relationships with environment and space

We used distance-based redundancy analysis (db-RDA) on each biological dissimilarity matrix to examine community–environment relationships in more detail [55]. This method is similar to redundancy analysis, but may be based on any dissimilarity or distance matrix (in our case, Bray-Curtis dissimilarity) [50].

Initially we selected only significant environmental and spatial predictors of variation in taxonomic and functional beta diversity, which then were used for the environmental model and spatial model [56]. We used forward selection with 9999 permutations to select the environmental variables to run the environmental model. For this, we used the matrices containing each beta diversity component and the environmental predictors and the MEMs. The selection stopped either when the tested variable had a *p*-value above 0.05 or when the adjusted *R*^*2*^ [56] of the full model, before any selection, was exceeded [56]. The forward selection procedure was run with the “forward.sel” function from the R package vegan [56]. The summary results of the forward selection procedure are presented in the Tables D and E in S1 File.

#### Variation partitioning for the anuran taxonomic and functional beta diversity

The relative contributions of the environmental descriptors and spatial variables to the taxonomic and functional beta diversity patterns were evaluated using a partial Redundancy Analysis (pRDA) with variation partitioning [51]. This analysis partitions the variance in community composition resulting from (1) each explanatory variable ([E] = environment and [S] = spatial), (2) the unique contribution of each explanatory variable ([E/S] = environment—purely environmental variables–or [S/E] = spatial–purely spatial variables) and (3) the total variance explained by the environmental and spatial variables together (spatially structured environmental variables). The variance explained by each fraction was based on the adjusted *R*^2^ [57].

The significance of db-RDA axes (Tables F and G in S1 File) and pRDA fractions of the selected models were tested through an ANOVA-like permutation test to assess the significance of the constraints, using 9999 permutations. The db-RDA and pRDA analyses were done using the functions “capscale” and “var.part”, and the permutations using the “anova.cca” function, of the R package vegan [58].

## Results

Eleven species belonging to three families (Bufonidae, Hylidae and Leptodactylidae) were registered. The most frequent species were *Dendropsophus sanborni* and *Pseudis minuta*, occurring in 19 of 33 ponds evaluated, respectively. *Physalaemus biligonigerus* and *Scinax fuscovarius* were less frequent occuring, respectively, in three and four of the sampled ponds (for the complete list of species and occurrence pattern across ponds see Table B in S1 File).

### Environmental and spatial predictors and anuran beta diversity

In general, the sets of descriptors selected to compose the environmental and spatial models were different between the taxonomic and the functional beta diversities (Table 2). The greater relationship between the selected environmental descriptors and beta diversity was observed in the total component of taxonomic beta diversity (*R*^2^*adj*: 0.18; *F*: 1.52; *p*: <0.001) and for turnover (*R*^2^*adj*: 0.18; *F*: 2.25; *p*: 0.02) and nestedness (*R*^2^*adj*: 0.29; *F*: 8.40; *p*: <0.001) components of functional beta diversity.

**Table 2:** Results of distance-based RDAs for the abundance data. Analyses were run for taxonomic and functional components based on total beta diversity, turnover and nestedness dissimilarities. Full models and marginal tests of significance for single environmental variables are shown (i.e., separate significance test for each variable in a model when all other terms are in the model).

TOTAL (βBray)			TURNOVER (βBal)			NESTEDNESS (βGra)		
Taxonomic beta diversity								
Overall test			Overall test			Overall test		
*R*^2^*adj*:0.18; *F*:1.52; *p*: <0.001			*R*^2^*adj*:0.08; *F*: 1.18; *p*< 0.001			*R*^2^*adj*:0.001; *F*: 0.99; *p*: 0.56		
Predictor Variable	*F*	*p*	Predictor Variable	*F*	*p*	Predictor Variable	*F*	*p*
Depth	1.83	0.03	Depth	2.41	0.02	Ins.veg	0.99	0.56
Ins.veg	1.42	0.01	Ins.veg	2.27	<0.001			
Subst.	1.20	0.01	Subst.	1.67	0.05			
Functional beta diversity								
Overall test			Overall test			Overall test		
*R*^2^*adj*:0.05; *F*: 1.84; *p*: 0.05			*R*^2^*adj*:0. 18; *F*: 2.25; *p*: 0.02			*R*^2^*adj*:0.29; *F*: 8.40; *p*: <0.001		
Predictor Variable	*F*	*P*	Predictor Variable	*F*	*p*	Predictor Variable	*F*	*P*
Margin veg.	1.84	0.05	Margin veg.	3.86	<0.001	Area	8.40	<0.001
			Subst.	1.46	0.20			

Regarding taxonomic beta diversity, the environmental descriptors selected were depth, pond vegetation and pond substrate. Pond vegetation was shared for all of beta diversity components, but it was not significant for the nestedness component. Depth and pond vegetation seem to the main drivers of taxonomic beta diversity in the metacommunity, since it largely explained total beta diversity (**depth**: *F* = 1.83, *p* = 0.03; **pond vegetation**: *F* = 1.42, *p* = 0.01) and turnover (**depth**: *F* = 2.41, *p* = 0.02; **pond vegetation**: *F* = 2.27, *p*<0.001; Table F in S1 File). The environmental descriptors selected for the functional beta diversity were area, vegetation around the pond and types of substrate. Vegetation around the pond was the descriptor that most explained total functional beta diversity (*F* = 1.84, *p* = 0.05; Table G in S1 File) and turnover (*F* = 3.86, *p*<0.001). For the nestedness, area was the descriptor that most explained total beta diversity of this component (*F* = 8.40; *p*< 0.001).

Spatial predictors (MEN) that significantly influenced beta diversity also varied according the facet of diversity. The predictors that influenced total taxonomic beta diversity were MEN 1, 3 and 14 (*R*^2^*adj*: 0.17; *p*: <0.001); all of these plus MEN 11, 12, 21 and 5 influenced taxonomic turnover, while MEN 7, 14 and 15 affected taxonomic nestedness (*R*^2^*adj*: 0.24; *p*: 0.01). The greatest influence of spatial predictors (MEN) was found on functional beta diversity. Total functional beta diversity responded mostly to MEN 12 and 22 (*R*^2^*adj*: 0.35; *p*: 0.01). MEN 12 plus 1, 4, 9, 13, 16, 18 and 21 were selected as the best predictors of the spatial model for the functional turnover (*R*^2^*adj*: 0.79; *p*: 0.01). Finally, functional nestedness was significantly influenced by MEN 2, 3, 12, 20 and 22 (*R*^2^*adj*: 0.71; *p*: 0.01).

### Variation partitioning for anuran taxonomic and functional beta diversity

**Fig 2:**
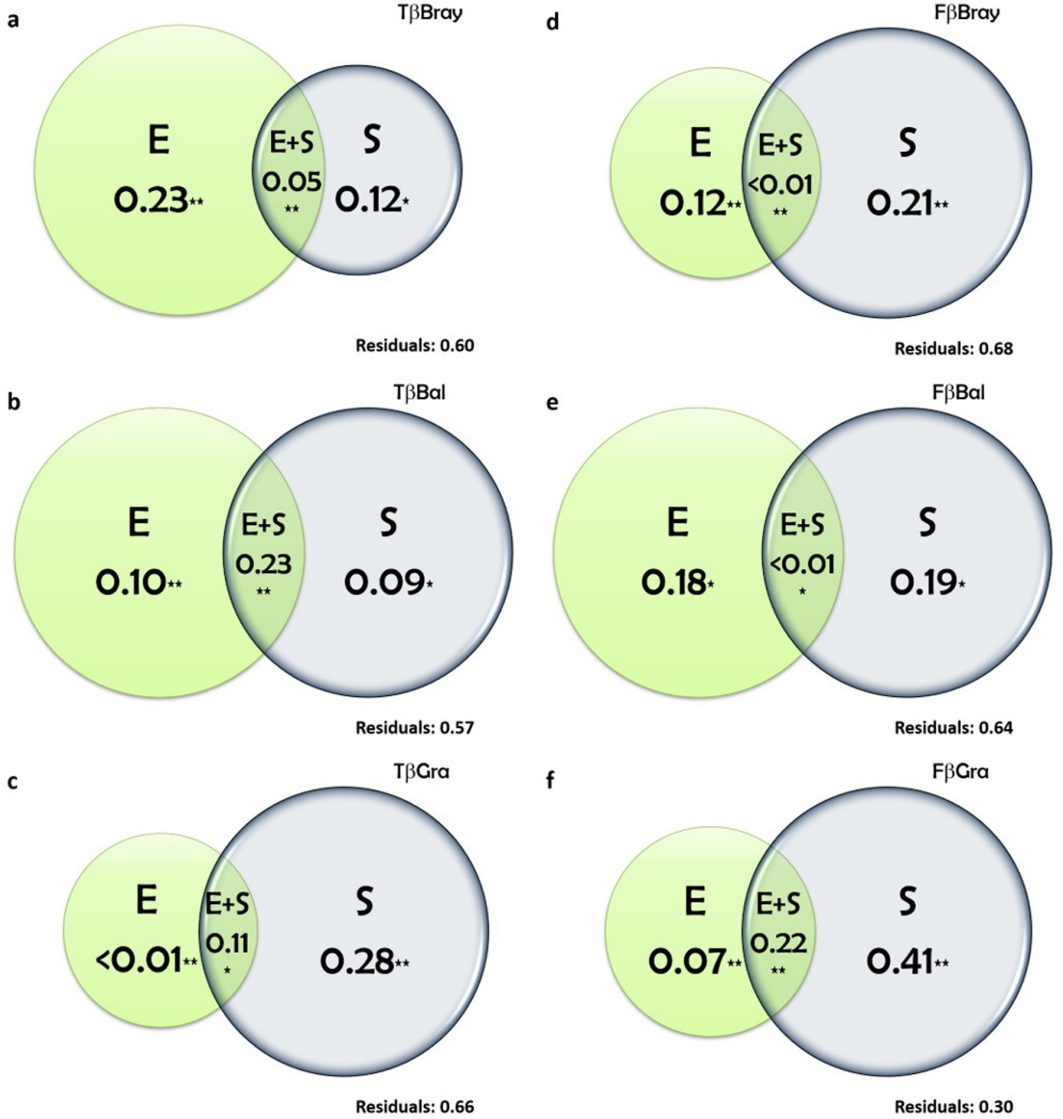
Variation partitioning for total taxonomic (a-c) and functional (d-f) beta diversities of anuran communities. E = environment; S = space. (*) *p*< 0.05; (**) *p*<0.001.

Variation partitioning analyses identified significant effects of both environmental and spatial components on the anuran metacommunity structure. Both environment and space influenced the taxonomic and functional structure, but the taxonomic structure was mostly driven by environment, while functional structure was mostly determined by the spatial component (Fig 2A–2E and Table H in S1 File).

The contribution of the environmental component for the total variance explained by the taxonomic beta diversity was greater than the pure spatial component (Fig 2A and Table H in S1 File). For total beta diversity, the contributions of the environmental individual fraction were 23%, while the contributions of space were 12%. The shared contribution between Environment and Space on the total variance was 5%.

The components of taxonomic beta diversity showed more complex patterns. The shared fraction between environment and space (11%), but mostly the pure spatial component (28%), explained nestedness patterns (**βGra; Fig 2C)**). The environmental component explained less than 1% of the variation (though it was significant; p = 0.004). The opposite pattern was observed for the turnover, where the shared fraction between environment and space presented the strongest influence on the patterns (**βBal** = 23%; Fig 2B). The environmental fraction was slightly larger than the spatial fraction in explaining the patterns of this component (**βGra** = 10% and 9%, respectively).

Variance in functional beta diversity (**βBray: 21%, Fig 2D)** and its components (**βBal: 19%, Fig 2E; βGra: 41%; Fig 2F))** were mostly explained by spatial processes (Table in S1 File). However, the environmental component also contributed significantly to the explained fraction, especially towards functional turnover (**βBal:**18%). Finally, the largest contribution of the shared fraction between environment and space was observed solely for the functional nesting. (**βGra**: 22%).

## Discussion

Here, we used a variation partition approach to understand how taxonomic and functional anuran beta diversity are influenced by environment and space at a regional scale in South American subtropical wetlands. Our results showed opposing patterns between taxonomic and functional beta diversity in their response to environmental and spatial predictors, contrary to what we had predicted, and has been described for metacommunities of Atlantic Forest anurans [30]. Taxonomic diversity responded to both spatial and local environmental predictors, while functional diversity was better explained by spatial predictors. We also registered opposite patterns for species turnover. While the spatially structured environment drives taxonomic turnover, the isolated fractions of space and environment were responsible for all the explanation found for functional turnover. However, it should be noted that although we found discrepancies between the taxonomic and functional structure, nestedness components for each were driven similarly by the same predictors (spatially structured environment and mostly spatial fraction).

Our findings reinforce that the structuring of beta diversity in metacommunities of organisms that depend on aquatic systems, including those of anurans, is complex [22, 30]. This allows us to infer that different processes act on the selection of species and functional attributes along metacommunities [22]. Also, the patterns and processes of diversity might differ when total beta diversity is partitioned [8]. Our results also support the assumption that beta diversity components vary across geographic space and may respond to different predictors [1, 12].

### Environmental predictors and anuran beta diversity

The organization of anuran assemblages in freshwater systems exhibits patterns in response to environmental gradients [29, 34, 59]. Most of the descriptors we evaluated had some level of influence on anuran patterns of beta diversity. Indeed, different environmental factors tend to affect species differently due to differences in their physiological and behavioural characteristics [20, 35, 38]. Area and depth were the two predictors best explaining beta diversity. These two variables are associated with pond hydroperiod and promote a trade-off between the persistence of those systems and predation and competition levels, both with strong influence in anuran survival and life cycle [31, 32]. Species richness and composition will thus be affected by those descriptors, and the persistence of a species in a given community will be mediated by the presence of specific traits [31, 32]. For example, species that lay eggs in foam nests and show rapid larval development (leptodactylids as *Physalaemus biligonigerus* and *Physalaemus gracilis*) survive in ephemeral ponds but tend to present reduced rates of growth and post-metamorphic survival [60, 61]. On the other hand, the occurrence in permanent ponds is favoured by morphological and behavioural traits that facilitate the escape and co-occurrence with predators and competitors, respectively (e.g. body and fin format; refuge use; activity patterns) at the cost of time delays in development and phenotypic changes promoted by intra and interspecific interactions [62].

Substrate type and pond and margin vegetation are closely associated with species' reproductive habits (e.g. calling sites and oviposition sites). Anurans present a wide variety of reproductive modes [63, 64], and the presence of more differentiated modes requires increasing levels of environmental complexity. This is the case for most species of the Hylidae and the Leptodactylidae, which place spawning near aquatic macrophytes [64]. These variables represent resources that were also crucial for adults and juveniles in their thermoregulation and food production processes [65, 66, 67]. The significant contribution of these variables in the metacommunity structure demonstrates the importance of environmental heterogeneity towards both taxonomic and functional structures. More specifically, our results demonstrate that the presence of ponds distributed along gradients promoted by substrate type and pond and margin vegetation is paramount for the maintenance of anuran populations and communities [32, 39, 68], and any change in these variables could drastically change the patterns of metacommunity organization.

### Opposing patterns between taxonomic and functional beta diversity

Our results show that taxonomic beta diversity is mainly structured by niche-based processes, as the similarity in species composition decays along the environmental gradient [12]. This means that environmentally distinct ponds add different community compositions [69, 70]. However, this dissimilarity was slightly lower for the functional diversity for most cases, indicating that, although there is a taxonomic turnover mediated by environmental filters, the traits present along these gradients are often not sufficiently different for large functional dissimilarities to be observed [25, 71].

Functional beta diversity was more closely associated with spatial predictors, suggesting the dominance of neutral processes [14]. The presence of a spatial structure in beta diversity patterns of anurans has been observed to communities of Amazonia [39] and southeastern Brazil [29, 38] and appears to be common and more evident for amphibians than for other organisms (e.g. mammals, birds and invertebrates) [1]. We highlight that the combination of spatial and environmental models also contributed considerably to the explained fraction, which may suggest a certain level of spatial autocorrelation in some of the environmental variables [3]. These diversity patterns organized according to spatially structured environmental variables may be common in freshwater communities [72]. This is because the ecological variables of ponds (e.g. depth and aquatic vegetation) are not spatially independent and are often spatially structured at local and regional scales [29, 72].

The results for taxonomic and functional nestedness emphasize the pattern of dominance by spatial predictors in our study area. This may be linked to dispersal limitation [36]; indeed, anurans relatively small body-size and physiological limitations make most species dependent on flooded or at least humid corridors for dispersal [20, 32]. In addition, the presence of natural and artificial barriers (e.g. sandy soils and roads with constant car traffic, as occurs in our study area) may prevent many species from reaching all ponds available for the whole of the metacommunity [1, 32]. As a consequence, there was a decrease in functional similarity with the increase in geographical distance, as occurs in other aquatic organisms [22, 73, 74].

Still, the association of spatial predictors with neutral dynamics and potential patterns of dispersal limitation should be interpreted cautiously. In fact, potentially important environmental variables may not have been evaluated, while contained in the spatial component [71, 75]. Also, other factors difficult to evaluate, such as predation and competition, may have a significant effect in the patterns of beta diversity and alter the importance of each predictor along the metacommunity, which was already demonstrated for microorganisms in controlled systems [76].

Processes of community assembly act in different environmental gradients and along spatial and temporal scales, resulting in patterns of functional and phylogenetic convergence or divergence independent of the taxonomic identity [27, 77]. For our anuran dataset taxonomic beta diversity responded to both environmental and spatial predictors, while functional beta diversity responded almost exclusively to space. While this differs from our predictions, similar discrepancies between metrics of diversity across spatial and temporal scales were already reported in other taxa [19, 78, 79, 80, 81].

## Conclusions

In conclusion, our results contributed to the knowledge about the relative influence of neutral and niche-based processes in determining the structure of anuran metacommunities. Along the southern coast of Brazil, the structure of beta diversity differed between the taxonomic and functional components. Despite this, we found important congruences between the components of beta diversity within each facet. The dominance of environmental and spatial predictors in the structuring of the taxonomic diversity and the spatial predictors in the structuring of the functional diversity suggest the co-existence of different processes in structuring anuran metacommunities and reinforce the importance of the inclusion of different facets of diversity in such analyses. The substantial contribution of purely spatial predictors in the patterns of both facets of diversity, confirms the great contribution of neutral processes on the structuring of anuran metacommunities [30]. However, the significant contribution of the shared fraction between environment and space, shows that the structure of our target metacommunity results from the interaction of both processes.

## Supporting information

S1 File Supporting data information. Table A—Environmental descriptors of ponds measured between October 2016 and March 2017 at Lagoa do Peixe Nation Park, Rio Grande do Sul, Brazil. (DOCX); Table B–Anuran species composition in each of the sampled ponds. (DOCX); Table C–Functional traits measured (in adults). (DOCX); Table D–Results for the forward selection of environmental variables to compose the spatial model of taxonomic and functional beta diversity during the pRDA analysis. (DOCX); Table E–Results of forward selection of spatial variables to compose the spatial model of taxonomic and functional beta diversity during the pRDA analysis. (DOCX); Table F–Results of anova.cca test for the two first axis of db-RDA between environmental variables selected and taxonomic beta diversity components;Table G–Results of anova.cca test for the two first axis of db-RDA between environmental variables selected and functional beta diversity components. (DOCX); Table H–Variation partitioning for the components of anuran beta diversity based on abundance data. The table shows the variation explained (*R*^*2*^ *adjusted*) for total taxonomic and functional beta diversity and turnover and nestedness compontens versus environment and space. E = environment; S = spatial component obtained from dbMEM; E+S = shared contribution between environment and space; E/S = the unique contribution of the environmental component; S/E = the unique contribution of the spatial component.
(DOCX)
