# Supplementary material for: Taxonomic and functional anuran beta diversity of a subtropical metacommunity respond differentially to environmental and spatial predictors
Source: PLoS One. 2019 Nov 14;14(11):e0214902. doi: 10.1371/journal.pone.0214902 (PMC6855460; doi:10.1371/journal.pone.0214902)
Supplement: S1 File — Table A—Environmental descriptors of ponds measured between October 2016 and March 2017 at Lagoa do Peixe Nation Park, Rio Grande do Sul, Brazil. (DOCX); Table B–Anuran species composition in each of the sampled ponds. (DOCX); Table C–Functional traits measured (in adults). (DOCX); Table D–Results for the forward selection of environmental variables to compose the spatial model of taxonomic and functional beta diversity during the pRDA analysis. (DOCX); Table E–Results of forward selection of spatial variables to compose the spatial model of taxonomic and functional beta diversity during the pRDA analysis. (DOCX); Table F–Results of anova.cca test for the two first axis of db-RDA between environmental variables selected and taxonomic beta diversity components;Table G–Results of anova.cca test for the two first axis of db-RDA between environmental variables selected and functional beta diversity components. (DOCX); Table H–Variation partitioning for the components of anuran beta diversity based on abundance data. The table shows the variation explained (R2 adjusted) for total taxonomic and functional beta diversity and turnover and nestedness compontens versus environment and space. E = environment; S = spatial component obtained from dbMEM; E+S = shared contribution between environment and space; E/S = the unique contribution of the environmental component; S/E = the unique contribution of the spatial component. (DOCX) [file pone.0214902.s001.docx]

# **Taxonomic and functional anuran beta diversity of a subtropical metacommunity respond differently to environmental and spatial predictors**

Dalmolin et al., 2019

## **S1 - SUPLLEMENTARY MATERIAL**

| **Table A –** Environmental descriptors of ponds measured between October 2016 and March 2017 at Lagoa do Peixe Nation Park, Rio Grande do Sul, Brazil. | | | | | | | | | | |  |
| --- | --- | --- | --- | --- | --- | --- | --- | --- | --- | --- | --- |
| **Environmental descriptors** | | **Description/levels** | | | **Ecological Relevance** | | | **Reference** | | | |
| Area | | Total surface area of the pond (m^2^) | | | Both are related to the occurrence and persistence of individuals in ponds, as well as the levels of competition and/or predation, and the reproductive success of each species | | | 9, 10 | | | |
| Depth | | Maximum depth of the pond (cm) | | |  |  |  |  |  |  |  |
| Distance to the nearest forest fragment | | Distance to the nearest forest fragment (m) | | | promote routes of dispersion and places for thermoregulation and feeding | | | 11 | | | |
| Distance to the nearest sampled pond | | Distance to the nearest other pond (m) | | | Ensure population persistence due to increased recolonization rates of ponds subject to stochastic extinctions. | | | 12 | | | |
| Pond vegetation | | Number of vegetation types inside the pond, according to the following categories their combinations: (i) absent; (ii) macrophytes; (iii) grasses; (iv) herbs; (v) shrubs; (vi) trees. | | | provides a greater variety of microhab-  itats in both vertical and horizontal strata and thus insures  that several species-specific requirements are met, promoting maintaining of a high diversity of  species | | | 11, 13 | | | |
| Margin configuration | | (i) flat border; (ii) angular border (margins with ≥15 cm higher than the surface water). | | | affects microclimatic conditions and foraging and reproductive success | | | 14 | | | |
| Vegetation around the pond | | \| Number of vegetation types around the pond, according to the following categories and their combinations:(i) grasses; (ii) herbs; (iii) shrub; (iv) trees. \| \| --- \| | | |  |  |  |  | | | |
| Pond substrate | | Number of substrate types of the pond, according to the following categories and combinations: (i) muddly; (ii) with vegetation. | | | influence habitat use and foraging of tadpoles | | | 14 | | | |
| \| **Table B –** Anuran species composition in each of the sampled ponds. \| \| \| \| \| \| \| \| \| \| \| \| \| \| \| \| \| --- \| --- \| --- \| --- \| --- \| --- \| --- \| --- \| --- \| --- \| --- \| --- \| --- \| --- \| --- \| --- \| \| **POND** \| *D. min.* \| *D. sanb.* \| *H. pul.* \| *L. lat.* \| *Ph. bil.* \| *Ps. fal.* \| *Ph. gra.* \| *Pse. min.* \| \| *R. dorb.* \| \| *Sc. fusc.* \| \| *Sc. squa.* \| \| \| **p1** \| 0 \| 21 \| 0 \| 4 \| 0 \| 0 \| 0 \| \| 1 \| \| 0 \| \| 0 \| \| 0 \| \| \| **P2** \| 2 \| 3 \| 0 \| 0 \| 0 \| 0 \| 0 \| \| 0 \| \| 0 \| \| 0 \| \| 0 \| \| \| **P3** \| 0 \| 0 \| 1 \| 2 \| 0 \| 0 \| 0 \| \| 0 \| \| 0 \| \| 1 \| \| 0 \| \| \| **p4** \| 0 \| 1 \| 4 \| 3 \| 0 \| 0 \| 1 \| \| 2 \| \| 0 \| \| 0 \| \| 0 \| \| \| **p5** \| 9 \| 0 \| 0 \| 0 \| 0 \| 0 \| 0 \| \| 0 \| \| 0 \| \| 0 \| \| 0 \| \| \| **P6** \| 0 \| 0 \| 0 \| 0 \| 0 \| 0 \| 0 \| \| 2 \| \| 0 \| \| 3 \| \| 1 \| \| \| **p7** \| 0 \| 20 \| 5 \| 0 \| 0 \| 0 \| 0 \| \| 0 \| \| 0 \| \| 0 \| \| 1 \| \| \| **p8** \| 0 \| 1 \| 6 \| 6 \| 0 \| 8 \| 4 \| \| 0 \| \| 0 \| \| 1 \| \| 8 \| \| \| **p9** \| 0 \| 0 \| 1 \| 5 \| 0 \| 1 \| 4 \| \| 2 \| \| 0 \| \| 0 \| \| 0 \| \| \| **p10** \| 0 \| 10 \| 0 \| 0 \| 0 \| 0 \| 1 \| \| 10 \| \| 0 \| \| 1 \| \| 10 \| \| \| **p11** \| 22 \| 12 \| 4 \| 6 \| 0 \| 0 \| 0 \| \| 0 \| \| 0 \| \| 0 \| \| 1 \| \| \| **p12** \| 10 \| 3 \| 0 \| 1 \| 0 \| 0 \| 14 \| \| 6 \| \| 0 \| \| 0 \| \| 1 \| \| \| **p13** \| 0 \| 24 \| 7 \| 2 \| 0 \| 0 \| 34 \| \| 9 \| \| 1 \| \| 0 \| \| 2 \| \| \| **p14** \| 0 \| 1 \| 6 \| 0 \| 0 \| 21 \| 6 \| \| 14 \| \| 2 \| \| 0 \| \| 1 \| \| \| **p16** \| 0 \| 23 \| 0 \| 1 \| 0 \| 0 \| 10 \| \| 1 \| \| 0 \| \| 0 \| \| 7 \| \| \| **p17** \| 0 \| 0 \| 3 \| 1 \| 0 \| 50 \| 10 \| \| 0 \| \| 2 \| \| 0 \| \| 0 \| \| \| **p18** \| 0 \| 0 \| 0 \| 7 \| 0 \| 0 \| 0 \| \| 0 \| \| 1 \| \| 0 \| \| 0 \| \| \| **P19** \| 2 \| 6 \| 0 \| 4 \| 0 \| 23 \| 8 \| \| 1 \| \| 0 \| \| 0 \| \| 0 \| \| \| **p20** \| 0 \| 0 \| 0 \| 0 \| 1 \| 0 \| 9 \| \| 0 \| \| 0 \| \| 0 \| \| 0 \| \| \| **p21** \| 0 \| 1 \| 0 \| 3 \| 1 \| 35 \| 6 \| \| 1 \| \| 1 \| \| 0 \| \| 0 \| \| \| **p23** \| 0 \| 1 \| 0 \| 1 \| 0 \| 0 \| 2 \| \| 1 \| \| 0 \| \| 0 \| \| 0 \| \| \| **P24** \| 0 \| 1 \| 9 \| 1 \| 12 \| 0 \| 0 \| \| 0 \| \| 0 \| \| 0 \| \| 0 \| \| \| **p25** \| 0 \| 7 \| 0 \| 2 \| 0 \| 1 \| 6 \| \| 0 \| \| 0 \| \| 0 \| \| 8 \| \| \| **p26** \| 0 \| 5 \| 21 \| 1 \| 0 \| 0 \| 6 \| \| 6 \| \| 1 \| \| 0 \| \| 0 \| \| \| **p27** \| 0 \| 0 \| 4 \| 0 \| 0 \| 0 \| 0 \| \| 1 \| \| 0 \| \| 0 \| \| 0 \| \| \| **p28** \| 6 \| 23 \| 30 \| 2 \| 0 \| 0 \| 11 \| \| 12 \| \| 0 \| \| 0 \| \| 23 \| \| \| **P29** \| 0 \| 0 \| 0 \| 0 \| 0 \| 14 \| 17 \| \| 53 \| \| 0 \| \| 0 \| \| 0 \| \| \| **p30** \| 2 \| 10 \| 0 \| 0 \| 0 \| 0 \| 20 \| \| 56 \| \| 0 \| \| 0 \| \| 5 \| \| \| **p31** \| 0 \| 0 \| 0 \| 0 \| 0 \| 0 \| 0 \| \| 3 \| \| 0 \| \| 0 \| \| 0 \| \| \| **p32** \| 0 \| 0 \| 5 \| 0 \| 0 \| 0 \| 0 \| \| 5 \| \| 0 \| \| 0 \| \| 0 \| \| \| **p33** \| 0 \| 0 \| 5 \| 0 \| 0 \| 0 \| 0 \| \| 0 \| \| 0 \| \| 0 \| \| 1 \| \|   *Dendropsophus minutus* (D. min.); *Dendropsophus sanborni* (D. sanb); *Hypsiboas pulchellus (H. pul.); Leptodactylus latrans (L. lat.); Physalaemus biligonigerus (Ph. bil.); Pseudopaludicola falcipes (Ps. fal.); Physalaemus gracilis (Ph. gra.); Pseudis minuta (Pse. min.); Rhinella dorbignyi (R. dorb.); Scinax fuscovarius (Sc. fusc.); Scinax squalirostris (Sc. squa.)*   \| **Table C -** Functional traits measured (in adults). \| \| \| \| \| --- \| --- \| --- \| --- \| \| Functional Trait \| Category \| Levels \| Reference \| \| Head shape \| continuous \| head length / head width \| This study \| \| Eyes position \| continuous \| Interorbital distance / head width \| This study \| \| Relative length of limbs \| continuous \| (Length of thigh + tibia length + tarsus length + foot length) / (arm length + forearm length + hand length) \| This study \| \| Eye size \| continuous \| Eye diameter / head length \| This study \| \| Body mass \| continuous \| grams \| This study \| \| Reproductive mode \| categorical \| From 1 to 40 \| 1 \| \| Relative number of eggs \| continuous \| number \| 2, 3, 4, 5, 6, 7, 8 \| \| Daily activity period \| categorical \| Diurnal; nocturnal; diurnal & nocturnal \| \| Type of habitat \| categorical \| Lentic; lotic; lentic & lotic \| \| Fossorial habit \| binary \| Present; absent \| \| Reproductive season \| categorical \| Dry; rain; dry & rain \|  \| **Table** D – Results for the forward selection of environmental variables to compose the environmental model of taxonomic and functional beta diversity during the pRDA analysis. \| \| \| \| \| \| \| \| \| --- \| --- \| --- \| --- \| --- \| --- \| --- \| --- \| \| Type of Data \| Beta Diversity Component \| Predictor Variable \| *R^2^* \| *adjusted R^2^ cumulated* \| *F* \| *p* \| \| **Taxonomic** \| **βBray** \| Depth \| 0.07 \| 0.03 \| 2.08 \| 0.04 \| \| Ins.veg \| 0.40 \| 0.16 \| 1.77 \| 0.005 \| \| Subst. \| 0.14 \| 0.20 \| 1.63 \| 0.05 \| \| **βBal** \| Depth \| 0.07 \| 0.03 \| 2.28 \| 0.03 \| \| Ins.veg \| 0.44 \| 0.20 \| 2.13 \| 0.005 \| \| Subst. \| 0.12 \| 0.25 \| 1.67 \| 0.05 \| \| **βGra** \| Ins.veg \| 0.01 \| 0.001 \| 1.28 \| 0.05 \| \| **Functional** \| **βBray** \| Margin veg. \| 0.10 \| 0.05 \| 2.44 \| 0.05 \| \| **βBal** \| Margin veg. \| 0.13 \| 0.08 \| 1.96 \| 0.05 \| \| Subst. \| 0.13 \| 0.10 \| 3.85 \| 0.005 \| \| **βGra** \| Area \| 0.33 \| 0.29 \| 8.40 \| 0.01 \| | | | | | | | | | |  |  |
| **Table E** – Results for the forward selection of spatial variables to compose the spatial model of taxonomic and functional beta diversity during the pRDA analysis. | | | | | | | | | | | |
| **TAXONOMIC BETA DIVERSITY** | | | | | | | | | | | |
| Facet of diveristy | Beta Diversity Component | | Predictor Variable | *R^2^* | | *adjusted R^2^ cumulated* | *F* | | *p* | | |
| **Taxonomic** | **βBray** | | MEM 1 | 0.10 | | 0.06 | 2.64 | | <0.001 | | |
|  |  |  | MEM 14 | 0.09 | | 0.12 | 2.35 | | 0.02 | | |
|  |  |  | MEM 3 | 0.08 | | 0.17 | 2.39 | | 0.02 | | |
|  | **βBal** | | MEM 1 | 0.10 | | 0.06 | 2.54 | | 0.01 | | |
|  |  |  | MEM 3 | 0.09 | | 0.12 | 2.46 | | 0.02 | | |
|  |  |  | MEM 11 | 0.08 | | 0.16 | 2.24 | | 0.04 | | |
|  |  |  | MEM 12 | 0.07 | | 0.21 | 2.20 | | 0.03 | | |
|  |  |  | MEM 21 | 0.07 | | 0.25 | 0.13 | | 0.04 | | |
|  |  |  | MEM 14 | 0.07 | | 0.30 | 2.22 | | 0.03 | | |
|  |  |  | MEM 5 | 0.06 | | 0.34 | 2.21 | | 0.03 | | |
|  | **βGra** | | MEM 14 | 0.12 | | 0.08 | 3.14 | | 0.02 | | |
|  |  |  | MEM 7 | 0.12 | | 0.17 | 3.41 | | 0.01 | | |
|  |  |  | MEM 15 | 0.10 | | 0.24 | 3.00 | | 0.01 | | |
| **Functional** | **βBray** | | MEM 22 | 0.24 | | 0.19 | 5.30 | | 0.01 | | |
|  |  |  | MEM 12 | 0.19 | | 0.35 | 5.27 | | 0.01 | | |
|  | **βBal** | | MEM 16 | 0.23 | | 0.19 | 5.15 | | 0.02 | | |
|  |  |  | MEM 1 | 0.13 | | 0.29 | 3.40 | | 0.04 | | |
|  |  |  | MEM 21 | 0.12 | | 0.39 | 3.54 | | 0.04 | | |
|  |  |  | MEM 9 | 0.13 | | 0.50 | 4.57 | | 0.02 | | |
|  |  |  | MEM 4 | 0.09 | | 0.59 | 4.12 | | 0.02 | | |
|  |  |  | MEM 12 | 0.09 | | 0.70 | 5.42 | | 0.006 | | |
|  |  |  | MEM 13 | 0.05 | | 0.75 | 3.44 | | 0.03 | | |
|  |  |  | MEM 18 | 0.04 | | 0.79 | 3.44 | | 0.04 | | |
|  | **βGra** | | MEM 22 | 0.31 | | 0.27 | 7.58 | | 0.01 | | |
|  |  |  | MEM 12 | 0.23 | | 0.48 | 7.75 | | 0.007 | | |
|  |  |  | MEM 20 | 0.09 | | 0.56 | 3.82 | | 0.04 | | |
|  |  |  | MEM 3 | 0.09 | | 0.64 | 4.26 | | 0.03 | | |
|  |  |  | MEM 2 | 0.07 | | 0.71 | 4.49 | | 0.02 | | |

| **Table** F – Results of anova.cca test for the two first axis of db-RDA between environmental variables selected and taxonomic beta diversity components. | | | | |
| --- | --- | --- | --- | --- |
| Type of Data | Beta Diversity Component | db-RDA Axis | *F* | *p* |
| Abundance | **βBray** | Axis 1 | 3.40 | 0.04 |
|  |  | Axis 2 | 2.88 | 0.26 |
|  | **βBal** | Axis 1 | 2.15 | 0.05 |
|  |  | Axis 2 | 1.88 | 0.43 |
|  | **βGra** | Axis 1 | 1.74 | 0.31 |
|  |  | Axis 2 | 1.05 | 0.99 |

| **Table G** – Results of anova.cca test for the two first axis of db-RDA between environmental variables selected and functional beta diversity components. | | | | |
| --- | --- | --- | --- | --- |
| Type of Data | Beta Diversity Component | db-RDA Axis | *F* | *p* |
| Abundance | **βBray** | Axis 1 | 1.99 | 0.05 |
|  | **βBal** | Axis 1 | 1.81 | 0.05 |
|  |  | Axis 2 | 0.94 | 0.95 |
|  | **βGra** | Axis 1 | 1.16 | 0.26 |
|  |  | Axis 2 | 0.84 | 0.83 |

| **Table H:** Variation partitioning for the components of anuran beta diversity based on **abundance** data. The table shows the variation explained (*R^2^ adjusted*) for total taxonomic and functional beta diversity and turnover and nestedness compontens versus environment and space. E = environment; S = spatial component obtained from dbMEM; E+S = shared contribution between environment and space; E/S = the unique contribution of the environmental component; S/E = the unique contribution of the spatial component. | | | | | | | |
| --- | --- | --- | --- | --- | --- | --- | --- |
|  |  | **TAXONOMIC** | | | **FUNCTIONAL** | | |
|  |  | R^2^adjusted | *F* | *p* | R^2^ adjusted | *F* | *P* |
| TOTAL BETA DIVERSITY **(βBray)** | E | 0.58 | 1.92 | <0.001 | 0.11 | 1.74 | 0.05 |
|  | S | 0.27 | 2.62 | <0.001 | 0.19 | 3.09 | 0.01 |
|  | E+S | 0.05 | 2.21 | <0.001 | <0.01 | 2.62 | 0.008 |
|  | E/S | 0.23 | 1.79 | <0.007 | 0.12 | 1.95 | 0.03 |
|  | S/E | 0.12 | 1.92 | 0.04 | 0.21 | 3.19 | <0.001 |
|  | Residuals | 0.60 | - | - | 0.68 | - | - |
| TURNOVER **(βBal)** | E | 0.34 | 2.16 | <0.001 | 0.17 | 2.26 | 0.02 |
|  | S | 0.32 | 2.77 | <0.001 | 0.19 | 1.47 | 0.05 |
|  | E+S | 0.24 | 2.07 | <0.001 | <0.01 | 1.91 | 0.02 |
|  | E/S | 0.10 | 1.37 | 0.04 | 0.18 | 1.96 | 0.05 |
|  | S/E | 0.09 | 1.26 | 0.05 | 0.19 | 1.69 | 0.05 |
|  | Residuals | 0.57 | - | - | 0.64 | - | - |
| NESTEDNESS **(βGra)** | E | 0.06 | 1.23 | 0.27 | 0.29 | 8.39 | <0.001 |
|  | S | 0.24 | 3.50 | 0.003 | 0.61 | 6.75 | <0.001 |
|  | E+S | <0.01 | 2.26 | 0.004 | 0.22 | 7.69 | <0.001 |
|  | E/S | 0.11 | 1.49 | 0.05 | 0.08 | 4.16 | 0.02 |
|  | S/E | 0.28 | 3.44 | 0.003 | 0.40 | 3.78 | <0.001 |
|  | Residuals | 0.60 | - | - | 0.30 | - | - |

# REFERENCES

1. Haddad C F B, Prado C P A. Reproductive modes in frogs and their unexpected diversity in the Atlantic forest in Brazil. BioScience. 2005; 55: 207-217.
2. Reinke M, Deiques C H. Natural history of *Hypsiboas leptolineatus* (Anura: Hylidae) in Aparados da Serra National Park, Rio Grande do Sul, Brazil. Neotropical Biology and Conservation. 2010; 5(3): 188-196. doi: 10.4013/nbc.2010.53.08
3. Camargo A, Naya D E, Canavero E, Rosa I da, Maneyro R.Seasonal Activity and the Body Size-Fecundity Relationship in a Population of Physalaemus gracilis (Boulenger, 1883) (Anura, Leptodactylidae) from Uruguay. Ann. Zool. Fennici. 2005; 42: 513–521.
4. Camargo A, Sarroca M, Maneyro R. Reproductive effort and the egg number vs. size trade-off in *Physalaemus* frogs (Anura: Leiuperidae). Acta Oecologica. 2008; 34: 163-171.
5. Duré M I, Schaefer E F, Hamann E I, Kehr A I. Consideraciones ecológicas sobre la dieta, la reproducción y el parasitismo de *Pseudopaludicola boliviana* (Anura, Leptodactylidae) de Corrientes, Argentina. Phyllomedusa. 2004; 3(2):121-131.
6. Melchiors J, Di-Bernardo M, Pontes G M F, de Oliveira R B, Solé M, Kwet A. Reprodução de Pseudis minuta (Anura, Hylidae) no sul do Brasil. 2004. Phyllomedusa. 2004; 3(1):61-68.
7. Pombal Jr. J P, Haddad C F B. Estratégias e modos reprodutivos de anuros (amphibia) em uma poça permanente na Serra de Paranapiacaba, sudeste do Brasil . Pap. avuls zool. 2005; 45(15)
8. Maneyro R, Carreira S. Guía de Anfibios del Uruguay. Montevideo, Ediciones de la fuga (Colección Ciencia Amiga); 2012.
9. Wellborn G A, Skelly D K, Werner E E. Mechanisms Creating Community Structure Across a Freshwater Habitat Gradient. Annual Review of Ecology and Systematics. 2002; 27(1): 337–363. https://doi.org/10.1146/annurev.ecolsys.27.1.337
10. Werner E E, Yurewicz K L, Skelly D K, Relyea R A. Turnover in an amphibian metacommunity: The role of local and regional factors. Oikos. 2007; 116(10): 1713–1725. https://doi.org/10.1111/j.0030-1299.2007.16039.x
11. Silva F R, Oliveira T A, Gibbs J P, Rossa-Feres D C. Na experimental assessment of landscape configuration effects on frog and toad abundance and diversity in tropical agro-savannah landscapes of southeastern Brazil. Landsc Ecol. 2012; 27:87–96
12. Hanski I. Metapopulation dynamics. Nature. 1998; 396: 41–49
13. Prado V H M, Rossa-Feres D C. Multiple Determinants of Anuran Richness and Occurrence in an Agricultural Region in South-Eastern Brazil. Environmental Management. 2014; 53: 823–837. DOI 10.1007/s00267-014-0241-y
14. Ribeiro J, Colli G R, Batista R, Soares A. Landscape and local correlates with anuran taxonomic, functional and phylogenetic diversity in rice crops. Landscape Ecology. 2017; 32(8): 1599–1612. https://doi.org/10.1007/s10980-017-0525-8
